# Curvilinear Association Between Language Disfluency and *FMR1* CGG Repeat Size Across the Normal, Intermediate, and Premutation Range

**DOI:** 10.3389/fgene.2018.00344

**Published:** 2018-08-24

**Authors:** Jessica Klusek, Anna Porter, Leonard Abbeduto, Tatyana Adayev, Flora Tassone, Marsha R. Mailick, Anne Glicksman, Bridgette L. Tonnsen, Jane E. Roberts

**Affiliations:** ^1^Department of Communication Sciences and Disorders, University of South Carolina, Columbia, SC, United States; ^2^Department of Psychology, University of South Carolina, Columbia, SC, United States; ^3^Department of Psychiatry and Behavioral Sciences, University of California, Davis, Sacramento, CA, United States; ^4^MIND Institute, University of California, Davis, Sacramento, CA, United States; ^5^Department of Human Genetics, New York State Institute for Basic Research in Developmental Disabilities, Staten Island, NY, United States; ^6^Department of Biochemistry and Molecular Medicine, University of California, Davis, Sacramento, CA, United States; ^7^Waisman Center, University of Wisconsin-Madison, Madison, WI, United States; ^8^Department of Psychological Sciences, Purdue University, Lafayette, IN, United States; ^9^Department of Psychology, University of South Carolina, Columbia, SC, United States

**Keywords:** fragile X carriers, *FMR1* premutation, verbal inhibition, executive dysfunction, language dysfluency, low-normal CGG repeats, gray zone, *FMR1* phenotype

## Abstract

Historically, investigations of *FMR1* have focused almost exclusively on the clinical effects of CGG expansion within the categories of the premutation (55–200 CGG repeats) and fragile X syndrome (>200 CGG repeats). However, emerging evidence suggests that CGG-dependent phenotypes may occur across allele sizes traditionally considered within the “normal” range. This study adopted an individual-differences approach to determine the association between language production ability and CGG repeat length across the full range of normal, intermediate, and premutation alleles. Participants included 61 adult women with CGG repeats within the premutation (*n* = 37), intermediate (i.e., 41–54 repeats; *n* = 2), or normal (i.e., 6–40 repeats; *n* = 22) ranges. All participants were the biological mothers of a child with a developmental disorder, to control for the potential effects of parenting stress. Language samples were collected and the frequency of language disfluencies (i.e., interruptions in the flow of speech) served as an index of language production skills. Verbal inhibition skills, measured with the Hayling Sentence Completion Test, were also measured and examined as a correlate of language disfluency, consistent with theoretical work linking language disfluency with inhibitory deficits (i.e., the Inhibition Deficit Hypothesis). Blood samples were collected to determine *FMR1* CGG repeat size. A general linear model tested CGG repeat size of the larger allele (allele-2) as the primary predictor of language disfluency, covarying for education level, IQ, age, and CGG repeats on the other allele. A robust curvilinear association between CGG length and language disfluency was detected, where low-normal (∼ <25 repeats) and mid-premutation alleles (∼90–110 repeats) were linked with higher rates of disfluency. Disfluency was not associated with inhibition deficits, which challenges prior theoretical work and suggests that a primary language deficit could account for elevated language disfluency in *FMR1-*associated conditions. Findings suggest CGG-dependent variation in language production ability, which was evident across individuals with and without CGG expansions on *FMR1.*

## Introduction

Compromised function of the *Fragile X Mental Retardation-1* (*FMR1*) gene has significant consequences for brain development and function (Darnell et al., 2011; Sidorov et al., 2013). *FMR1* gene function is directly tied to the length of a trinucleotide (CGG) repeat on the 5’untranslated region (5′UTR) (Chen et al., 2003; Peprah et al., 2010). Historically, investigations of the *FMR1* phenotype have focused almost exclusively on two clinical conditions associated with CGG expansion: fragile X syndrome and *FMR1* premutation associated disorders. In most cases, fragile X syndrome occurs when a “full mutation" expansion > 200 CGG repeats occurs, leading to gene silencing and failure to produce Fragile X Mental Retardation Protein (FMRP). Fragile X syndrome is the most common form of inherited intellectual disability; yet, it is relatively rare, affecting 1:4,000–8,000 individuals (Song et al., 2003; Coffee et al., 2009; Santoro et al., 2012). In contrast, the *FMR1* premutation, defined by a CGG expansion in the 55–200 CGG repeat range, is exceedingly common, occurring in 1:291 females and 1:855 males (Hunter et al., 2014). Individuals with premutation alleles are at risk for a range of cognitive, language, social, affective, and physical health symptoms, which vary in penetrance and severity (Wheeler et al., 2017). In addition, female and male premutation carriers can develop a late onset neurodegenerative disorder named fragile X-associated tremor/ataxia syndrome (FXTAS), and approximately 20% of females will develop fragile X-associated primary ovarian insufficiency (FXPOI; Rodriguez-Revenga et al., 2009; Sullivan et al., 2011). These clinical features are thought to result from mild reductions in FMRP, elevated levels of messenger RNA (mRNA), and repeat-associated non-AUG (RAN) translation that occur in individuals with CGG expansions within the premutation range (Hagerman and Hagerman, 2013; Todd et al., 2013).

The boundary that separates the *FMR1* premutation from the “normal” repeat range is not clear-cut. Clinical involvement has been reported, albeit not in all studies, at the “intermediate” CGG repeat range (41–54 repeats), leading to coinage of the term “gray zone” to describe this CGG region of unclear clinical significance that bridges the boundaries of “normal” and “premutation” (Bretherick et al., 2005; Loesch et al., 2009, 2011; Hall et al., 2011, 2012; Liu et al., 2013; Debrey et al., 2016). These clinical findings are supported by molecular genetic evidence demonstrating a continuous scale of increased *FMR1* mRNA across premutation and intermediate alleles (Kenneson et al., 2001; Chen et al., 2003; Loesch et al., 2007; Sellier et al., 2014). Indeed, it has been suggested that the translational efficiency of *FMR1* is optimized when CGG repeats are the population mode, which has been documented at 29 or 30 copies (Chen et al., 2003; Ludwig et al., 2011; Tassone et al., 2011; Peprah, 2012; Kraan et al., 2018). Specifically, some evidence suggests increased protein translation when *FMR1* CGG repeat sizes are at the modal number, relative to higher or lower CGG repeat lengths (Chen et al., 2003). Therefore, it is possible that CGG repeats that deviate from the population mode may be associated with inefficient translation and possible adverse phenotypic outcomes, despite being within the “normal” range. Consistent with this hypothesis that *FMR1* gene function varies across the range of CGG repeat length, emerging evidence suggests “low-normal” CGG repeat numbers have been linked with cognitive difficulties, cancer risk, and increased likelihood of having a child with a disability (Weghofer et al., 2012; Mailick et al., 2014b, 2017; Adamsheck et al., 2017). In these reports, “low-normal” repeats have been variably defined as ≤22, ≤23, or ≤25 CGG repeats and have typically been captured through dichotomous grouping of males who possess a low-normal allele or females who are homozygous for low-normal alleles on both X chromosomes (Mailick et al., 2014a; Adamsheck et al., 2017). The findings of Mailick et al. (2017) also suggest that gene-environment interactions may play a role in the manifestation of risk at low-normal CGG repeats. In a population-based study of over 5,000 parents, low-normal alleles were associated with poorer health and functional outcomes only among parents who had a child with a disability, suggesting that the low-normal genotype may have made parents more vulnerable to environmental stressors.

Outside of these few reports very little is known about phenotypic variation occurring across the continuous range of normal CGG repeats (i.e., 6–40 copies) in the general population. Together, this emerging body of research suggests that CGG-dependent phenotypes may occur across the range of normal, intermediate, and premutation alleles, with the categorical demarcation of these boundaries being less straightforward than once assumed. Expanding on this work, the present study adopted a continuous approach to identify CGG-dependent phenotypes occurring across the full range of normal, intermediate, and premutation alleles. We focused specifically on language fluency because it is a feature that can be measured continuously, displays inter-individual variability in the general population, and accurately discriminates individuals with the *FMR1* premutation from controls (i.e., Movaghar et al., 2017).

Language disfluency is defined by interruptions in the flow of speech, such as revisions, repetitions, and fillers (e.g., “um”). These features are thought to reflect disruptions in utterance planning and production resulting from both language-specific processes (e.g., slow lexical retrieval) as well as executive problems that can manifest through linguistic function, such as deficits in planning or inhibition (Kemper et al., 2001; Burke et al., 2008). High rates of disfluency are seen in a number of neurocognitive disorders, such as attention-deficit/hyperactivity disorder and Alzheimer’s disease (Engelhardt et al., 2011; López-de-Ipiña et al., 2013). Disfluencies also occur in the speech of neurotypical individuals, affecting about 6% of words on average (Bortfeld et al., 2001). Recent evidence indicates that mothers with premutation alleles exhibit elevated rates of disfluency, with the presence of these disfluencies accurately discriminating mothers with premutation alleles from mothers of children with other developmental disorders (Sterling et al., 2013; Movaghar et al., 2017). In this emerging work, the severity of disfluency was not correlated with CGG size within the premutation range (Sterling et al., 2013), although non-linear associations was not tested. Follow-up studies are needed to test curvilinear CGG associations, which have been detected relative to other aspects of the *FMR1* premutation phenotype (Ennis et al., 2006; Roberts et al., 2009; Seltzer et al., 2012; Mailick et al., 2014a).

The cognitive-executive mechanisms underlying disfluency are also unclear. Disfluency among carriers of the *FMR1* premutation is thought to stem from the underlying executive deficits that are characteristic of this group (Sterling et al., 2013). This assumption has not been tested empirically, although emerging reports within the broader psycholinguistics literature support a link between disfluency and individual differences in executive function (Turkstra et al., 2004; Engelhardt et al., 2013). Theoretical work suggests that inhibitory aspects of executive control, specifically, plays a role in language disfluency. According to this Inhibition Deficit Hypothesis, inhibition deficits prevent irrelevant information from being filtered from working memory, leading to “mental clutter” and the inability to suppress inappropriate words and word sequences during language production (Hasher and Zacks, 1988). The hypothesized role of inhibition in language disfluency is particularly relevant to the study of *FMR1*, given that inhibition deficits have been documented in individuals with the *FMR1* premutation (Yang et al., 2013; Kraan et al., 2014b; Shelton et al., 2014). In sum, an inhibition deficit is a plausible explanation for *FMR1*–associated language disfluency, although this hypothesis has yet to be tested.

In summary, growing evidence suggests that CGG-dependent phenotypic variation may occur across the continuous range of normal, intermediate, and premutation *FMR1* alleles. However, genotype–phenotype associations across the full range of CGG repeat sizes are poorly understood because investigations of *FMR1* have traditionally focused on clinical conditions of the *FMR1* premutation and fragile X syndrome. The present study examined language disfluency as a sensitive linguistic marker that may relate to variation in CGG repeat length across individuals with and without CGG expansions. We adopted a continuous individual-differences approach to determine CGG-dependent changes in language fluency across normal, intermediate, and premutation allele sizes. Building on prior theoretical work, we also investigated inhibitory control as an executive process that may relate to language fluency, and may also be associated with CGG repeat size. Our specific research questions were: (a) What is the relationship between language disfluency and verbal inhibition skills? and (b) Is CGG repeat length associated with language disfluency and verbal inhibition skills across the continuous range of normal, intermediate, and premutation alleles?

## Materials and Methods

### Participants

The study cohort included 61 adult females, aged 26.6–64.2 years (*M* = 45.8, *SD* = 8.6). To control for the potential effects of parenting stress related to raising a child with a disability, all participants were biological mothers of a child with a neurodevelopmental disorder (the mean child age was 16.7 years, *SD* = 6.5). Twenty-four participants had a child with autism spectrum disorder (ASD) and CGG repeat sizes within the normal range of 6–40 repeats (*n* = 22) or the intermediate range of 41–54 repeats (*n* = 2). We defined the intermediate range as 41–54 repeats, following Loesch et al. (2007) and Hall et al. (2011). Thirty-seven participants had a child with fragile X syndrome and carried premutation alleles of 55–200 CGG repeats. All participants had a full scale IQ ≥ 80 on the Kaufman Brief Intelligence Test-2 (Kaufman and Kaufman, 2004), with a mean full scale IQ of 104.7 (*SD =* 12.3). Participants were native speakers of American English and clinical fluency disorders (e.g., stuttering) were ruled out via observation by a speech-language pathologist during administration of the study protocol. No participants reported a clinical diagnosis of Fragile X Associated Tremor Ataxia Syndrome (FXTAS). The sample primarily identified as Caucasian (86%) or African American (8%). Most participants had completed some college (33%), a bachelor’s degree (25%), or a graduate degree (27%). Detailed descriptive statistics are reported in **Table 1**.

**Table 1:** Descriptive statistics.

Variable	CGG Repeat Category (Allele-2)					

	Low-normal (≤25)	Normal (26–32)	High-normal (33–40)	Intermediate (41–54)	Premutation-low (55–89)	Premutation-mid (90–110)	Premutation-high (111–200)

*n*	3	13	6	2	14	19	4
**Allele-1 CGG size**							
*M* ±*SD*	21.0 ± 1.7	27.2 ± 5.94	29.2 ± 3.4	30.0 ± 0	29.4 ± 5.2	28.1 ± 3.6	29.0 ± 0.8
Range	20.0–23.0	10.0–31.0	23.0–33.0	30.0–30.0	20.0–43.0	20.0–32.0	28.0–30.0
**Age**							
*M* ±*SD*	49.4 ± 10.5	44.2 ± 8.7	40.2 ± 10.3	56.1 ± 2.7	46.3 ± 7.7	46.8 ± 8.2	44.6 ± 11.2
Range	37.5–57.4	31.9–62.1	29.4–56.5	54.2–58.0	26.6–60.0	30.6–64.18	30.4–56.8
**IQ**							
*M* ±*SD*	113.1 ± 10.4	101.8 ± 11.2	100.4 ± 15.8	97.5 ± 7.8	107.8 ± 14.0	103.9 ± 11.9	110.0 ± 7.8
Range	102.2–123.0	83.0–126.0	86.0–127.0	92.0–103.0	88.0–130.0	81.0–126.0	101.0–117.0
**Overall disfluency percent**							
*M* ±*SD*	9.3 ± 2.2	6.7 ± 2.6	8.2 ± 2.3	7.9 ± 4.2	6.9 ± 2.1	9.1 ± 2.6	8.5 ± 2.1
Range	7.8–11.9	3.0–11.9	5.4–12.4	5.0–10.9	3.4–9.9	3.3–12.8	5.7–10.5
**Repetition disfluency percent**							
*M* ±*SD*	1.3 ± 1.7	1.0 ± 0.6	1.2 ± 0.9	1.2 ± 0.9	1.0 ± 0.5	1.2 ± 0.9	1.3 ± 0.5
Range	0.1–3.3	0–3.1	0–2.6	0.5–1.8	0.3–2.0	0.2–3.5	0.7–1.8
**Repair disfluency percent**							
*M* ±*SD*	1.9 ± 0.5	1.5 ± 0.7	0.9 ± 0.6	1.6 ± 0.2	1.4 ± 0.5	1.5 ± 0.7	1.7 ± 0.7
Range	1.3–2.1	0.3–2.8	0.3–1.8	1.4–1.7	0.6-2.5	0.3–2.8	1.1–2.7
**Filled pause disfluency percent**							
*M* ±*SD*	6.1 ± 0.8	4.6 ± 1.8	6.0 ± 1.8	5.2 ± 3.5	4.5 ± 2.1	6.1 ± 2.0	5.5 ± 2.2
Range	5.3–6.8	1.8–8.0	4.2–9.1	2.7–7.6	0.2–8.1	2.4–9.9	3.6–8.0
**Hayling error score**							
*M* ±*SD*	3.0 ± 1.4	5.8 ± 6.9	4.8 ± 5.3	19.5 ± 17.7	12.2 ± 10.6	8.6 ± 11.9	4.3 ± 2.5
Range	2.0–4.0	0–23.0	1.0–14.0	7.0–32.0	1.0–33.0	0–43.0	1.0–7.0
**Hayling error score log transformation**							
*M* ±*SD*	1.4 ± 0.4	1.5 ± 1.0	1.5 ± 0.8	2.8 ± 1.0	2.2 ± 1.0	1.7 ± 1.1	1.5 ± 0.6
Range	1.1–1.6	0–3.2	0.7–2.7	2.1–3.5	0.7–3.5	0–3.8	0.7–2.1

Assessments took place as part of a larger study of social communication in the *FMR1* premutation, which has been previously described (e.g., Klusek et al., 2017a). Participants were recruited through their children who were participating in ongoing developmental studies of fragile X syndrome or ASD (e.g., Hogan et al., 2017; Matherly et al., 2018) or from the local community via social media, flyers, and word-of-mouth recruitment strategies targeting mothers of children with fragile X syndrome or ASD. Local recruitment methods included social media, word of mouth, and flyers posted at local pediatrician offices. Written informed consent was obtained and study procedures were approved by the Institutional Review Board of the University of South Carolina and conducted in accordance with the Declaration of Helsinki.

### Language Disfluency

Disfluencies were evaluated from language produced during the Five Minute Speech Sample (FMSS; Magaña et al., 1986). The FMSS is a language sample in which participants talk about “what kind of person” their child is and “how they get along” with their child for 5 min without any interruptions from the examiner. The FMSS provides an ideal sample of spontaneous, non-interrupted language from which to code disfluencies and has been used as the basis for disfluency coding in prior research on the premutation phenotype (Sterling et al., 2013; Movaghar et al., 2017). Samples were transcribed using Systematic Analysis of Language Transcripts (Miller and Chapman, 2008) conventions by blinded research assistants trained to 85% morpheme-morpheme agreement on three consecutive training files. Twenty percent of transcripts were randomly selected for inter-rater reliability by an independent transcriber, with average morpheme-to-morpheme agreement at 92%. Transcripts were coded for filled pauses, repetitions, revisions, and false starts, following the definitions outlined by Dollaghan and Campbell (1992) and Thurber and Tager-Flusberg (1993). Filled pauses were defined as non-lexical filler vocalizations (e.g., “um”) and lexical fillers (e.g., “you know”). Repetition disfluencies consisted of identical repetitions of a unit at the partial-word, word, or utterance level (e.g., “he he went to the store.”). Repairs consisted of revisions (modification of a unit already produced by the speaker, such as “he she went to the store.”) and false starts (utterances that are abandoned/not brought to a successful, coherent conclusion and do not involve any attempt to correct an error or add, delete, or change information, such as “he went to… I never met his teacher.”). The total number of disfluencies were tallied and divided by the total number of words to control for the amount of talk. Disfluencies were coded by two raters who were naïve to genetic status and were trained to *κ* ≥ 0.85 reliability with each other prior to independent coding. Inter-rater reliability estimated on 20% of random transcripts was estimated at *κ* = 0.85 for total disfluencies, 0.95 for repetitions, 0.85 for repairs, and 0.99 for filled pauses, indicating “outstanding” agreement across all categories (Landis and Koch, 1977).

### Verbal Inhibition

Verbal inhibition was measured with the Hayling Sentence Completion Test (Burgess and Shallice, 1997). In this test the examiner reads two sets of 15 sentences that have the last word missing. In the first set the participant provides a word that completes the sentence as quickly as they can. In the second set the participant completes the sentence with an unconnected word as quickly as possible, thus requiring the inhibition of an established prepotent response. Responses from the second set are scored for category A errors (responses that are connected to the sentence) and category B errors (responses that are loosely connected to the sentence). The converted A + B error score was computed as described by the test developers. Potential converted error scores range from 0 to 78 and higher scores reflect inhibitory errors that are more frequent and/or severe. Impaired performance on this index has been previously documented in women with the *FMR1* premutation (Kraan et al., 2014b).

### FMR1 CGG Repeat Number

CGG repeat size analysis of the 5′-UTR of *FMR1* was conducted on either DNA isolated from peripheral blood lymphocytes using standard methods (Qiagen, Valencia, CA, United States) or extracted from whole blood dried blood spots, as previously described (Adayev et al., 2014). Polymerase chain reaction (PCR) amplification of the *FMR1* CGG repeat region was conducted with AmplideX^®^ FMR1 PCR (RUO) reagents according to the manufacturer’s directions (Asuragen, Austin, TX 78744, United States). PCR products were analyzed by capillary electrophoresis and GeneMapper software for *FMR1* allele CGG repeat sizing (ABI 3130 Genetic Analyzer, Applied Biosystems, Foster City, CA, United States) (Chen et al., 2010). DNA analysis was conducted at either the MIND Institute at the University of California, Davis or the New York State Institute for Basic Research in Developmental Disabilities. To evaluate inter-lab reliability, 18% of participants submitted blood samples to both labs. Intraclass correlation coefficients (ICC [3,1]) indicated excellent reliability at 0.99 for both alleles. The allele with the lower CGG repeat length was designated allele-1 and the one with the higher repeat length as allele-2, consistent with the terminology used in prior reports (e.g., Gleicher et al., 2009a; Voorhuis et al., 2014).

### Statistical Analysis

Analyses were conducted in SAS v9.4 (SAS Institute, 2013). Descriptive statistics were computed and variables were examined for normalcy. The Hayling Error Score was log transformed to correct for right skew and the transformed variable was used in all analyses. To test the first research question regarding the association between disfluency and verbal inhibition, we computed Pearson correlations between the disfluency variables and the Hayling error score. Next, we fit general linear models to test CGG size as a predictor of disfluency and the Hayling error score. Examination of the plotted raw data suggested non-linear patterns and therefore quadratic and cubic terms for CGG repeat size were probed in the models. The models were successively fit with higher-order polynomial terms. If the polynomial term accounted for significant variance and visual examination of the fit diagnostic plots indicated improved model fit relative to the lower-order model, the higher-order term was retained. In all models we focused on CGG repeat size on allele-2 (the allele with the higher copy number) as the primary predictor and corrected for CGG repeat size on the other allele statistically. Although there are a variety of possible analytic methods to account for the presence of two X chromosomes in females, there is little consensus in the field as to which analytic technique is optimal. We selected this analytic technique based on established precedent, as this is the most common method used to account for two alleles in females in the extant literature (Gleicher et al., 2009a,b; Lledo et al., 2012; Voorhuis et al., 2014; Schufreider et al., 2015). We did not have activation ratio data available (i.e., the percent of cells carrying any given allele on the active X chromosome), which prevented us from employing other techniques that account for the relative influence of each allele (i.e., Allen et al., 2004). In addition to the CGG length on allele-1, covariates included education level, IQ, and age, which have been linked to individual differences in disfluency in prior work (e.g., Bortfeld et al., 2001; Engelhardt et al., 2013; Sterling et al., 2013). We retained the same covariates in the Hayling model to facilitate comparison across the models. Education level was coded as a four-level categorical variable reflecting the highest degree of educational attainment (grade school, high school, associate’s/bachelor’s degree, graduate degree). Age and IQ were grand-mean centered to facilitate interpretation of the parameter estimates. Partial eta squared ($\eta_{p}^{2}$) effect sizes were computed, with values of 0.01, 0.06, and 0.14 reflecting “small,” “medium,” and “large” effects, respectively (Cohen, 1988). To facilitate comparison with prior literature focused on the *FMR1* premutation, the general linear models were also repeated while limiting the sample to individuals with premutation alleles.

## Results

### Descriptive Statistics

The number of CGG repeats was treated as a continuous variable in all inferential analyses, however, for descriptive purposes we constructed categories based on CGG repeat length. The demarcation of “low-normal” and “high-normal” alleles has not been established in the literature. For descriptive purposes, we defined low-normal at ≤25 repeats and set the boundary for high-normal at ≤33 repeats, consistent with Gleicher et al. (2010). Low, mid-size, and high premutation alleles were categorized as 55–89, 90–110, 111–200, consistent with the zone of mid-size vulnerability detected by Mailick et al. (2014a). Consistent with our analytic approach (described above), descriptive characterization of the participants was based on allele-2 (the larger allele). Descriptive statistics are presented in **Table 1**. Three participants had low-normal repeats on allele-2; it is notable that these participants also had low-normal repeat sizes on allele-1 and thus were homozygous for low-normal repeats on both alleles. Across all participants, low-normal repeats on allele-1 occurred relatively frequently: 26% of individuals with normal/intermediate repeats on allele-2 and 22% of individuals with premutation repeats on allele-2 had low-normal repeats on the smaller allele. The cohort had relatively limited representation of individuals with intermediate CGG repeats on allele-2 (*n* = 2), which is a limitation (see *Discussion*). For descriptive purposes, performance on the disfluency and inhibition variables was compared across dichotomized groups of individuals with normal vs. premutation alleles. Individuals with a premutation allele showed increased repair disfluencies relative to those with normal alleles (*p* = 0.049). No other differences were observed across these dichotomized groups in repetition disfluencies (*p* = 0.461), filled pause disfluencies (*p* = 0.719), overall disfluency (*p* = 0.307), and Hayling error score (*p* = 0.172).

### Relationship Between Language Disfluency and Verbal Inhibition

Pearson correlations tested the association between disfluency and verbal inhibition. The Hayling error score was associated with severity of repair disfluencies (*r* = -0.31, *p* = 0.020) but not with overall disfluency (*r* = -0.17, *p* = 0.211) or the repetition or filled pause disfluency subtypes (*r* = 0.01, *p* = 0.950; *r* = -0.12, *p* = 0.374, respectively). Patterns were similar when correlations were tested within the subgroup of individuals with premutation alleles: a significant correlation was observed with repair disfluencies (*r* = -0.45, *p =* 0.006) but not with the other disfluency variables (all *r*’s < -0.18, *p*’s > 0.295).

### CGG Repeat Size as a Predictor of Language Disfluency

#### Overall Disfluency

**FIGURE 1:**
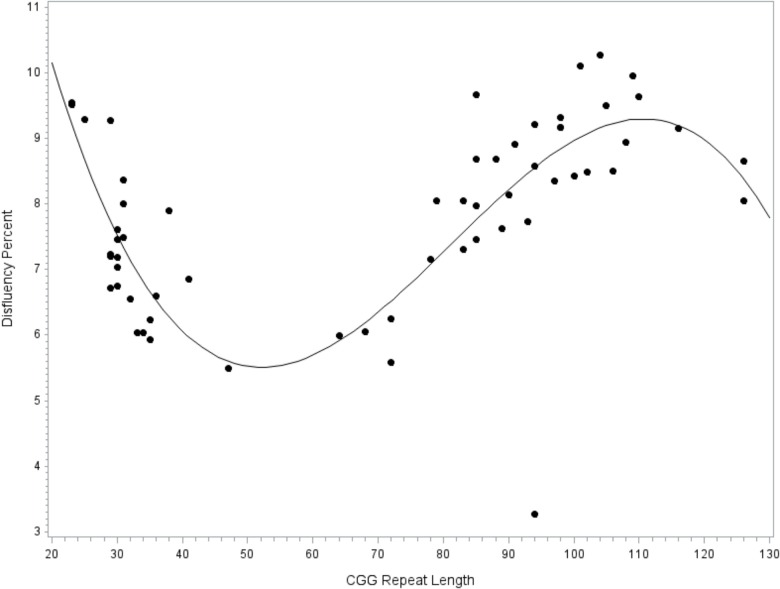
Relationship between disfluency and CGG repeat length. Cubic association between CGG repeat length and disfluency. Model-predicted values are shown, controlling for education level, IQ, age, and allele-1 CGG size.

A general linear model tested CGG repeat length as a predictor of disfluency. Regression diagnostics indicated that one case had an unduly large influence on the regression coefficients, as indicated by a Cook’s *D* value that far exceeded the recommended cut-off criteria (i.e., *D*_i_ > 4/*n*-*k*-1; Cook, 1977) and was considerably larger than all other cases (*D =* 1.34, all other *D*’s < 0.13). This highly influential point was excluded from the final model. The final overall model accounted for significant variance in total disfluency, *F*(9,49) = 2.40, *p* = 0.024, *R*^2^= 0.31. Significant effects were observed for the linear (*p* = 0.013, $\eta_{p}^{2}$ = 0.12), quadratic (*p* = 0.014, $\eta_{p}^{2}$ = 0.12), and cubic terms (*p* = 0.018, $\eta_{p}^{2}$ = 0.11) for allele-2 CGG size, with medium-to-large effect sizes. Disfluencies were the least likely in individuals with 40–70 repeats, increased by degree in individuals with 70–110 repeats, then decreased at > 110 repeats; see **Figure 1**. An increase in disfluency was also observed at <30 CGG repeats. The terms for education level, age, IQ, and allele-1 CGG size did not contribute significantly to the model (all *p’s* > 0.213).

**FIGURE 2:**
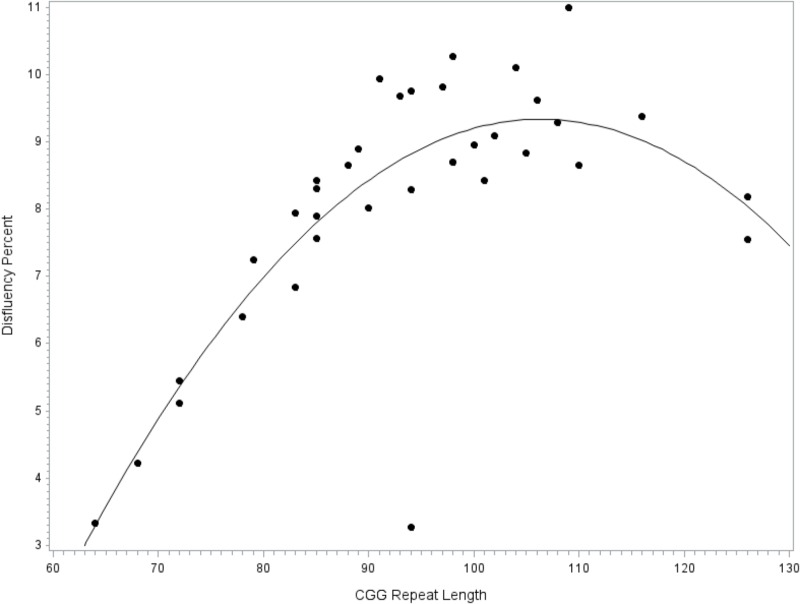
Relationship between language disfluency and CGG repeat length across the premutation range. Quadratic association between CGG repeat length and disfluency. Model-predicted values are shown, controlling for age, education level, IQ, and allele-1 CGG size.

To facilitate comparison with extant literature focused on the *FMR1* premutation, the model was repeated while restricting the sample to the subset of participants with premutation alleles. Similar to the full model reported above, fit diagnostics indicated that one observation far exceeded the recommended Cook’s *D* cut-off criteria for influence and this point was excluded. The overall model accounted for significant variance in disfluency, *F*(8,27) = 4.02, *p* = 0.003, *R*^2^= 0.54. The covariates for education level (*p* = 0.077), age (*p* = 0.336), IQ (*p* = 0.293), and allele-1 CGG repeat size (*p* = 0.435) did not contribute significantly to the model. Significant linear (*p* = 0.002, $\eta_{p}^{2}$ = 0.29) and quadratic (*p* = 0.005, $\eta_{p}^{2}$ = 0.26) terms for CGG size were detected; see **Figure 2**.

#### Disfluency Subtypes

Associations with specific disfluency subtypes were explored. The model predicting filled pause disfluency yielded results that were nearly identical to the overall disfluencies model. The cubic model was the best fit, with significant linear, quadratic, and cubic terms for CGG size, of medium-to-large effect sizes (all *p*’s < 0.018, $\eta_{p}^{2}$’s > 0.11). The curvilinear pattern with filled pause disfluencies mirrored that of the overall disfluency model, which is depicted in **Figure 1**. Filled pauses disfluencies were increased at <30 and 70–110 CGG repeats, and the lowest at 40–70 repeats. No significant linear, quadratic, or cubic effects for CGG repeat length were observed in the models predicting repair or repetition disfluency subtypes.

### CGG Repeat Size as a Predictor of Verbal Inhibition

**FIGURE 3:**
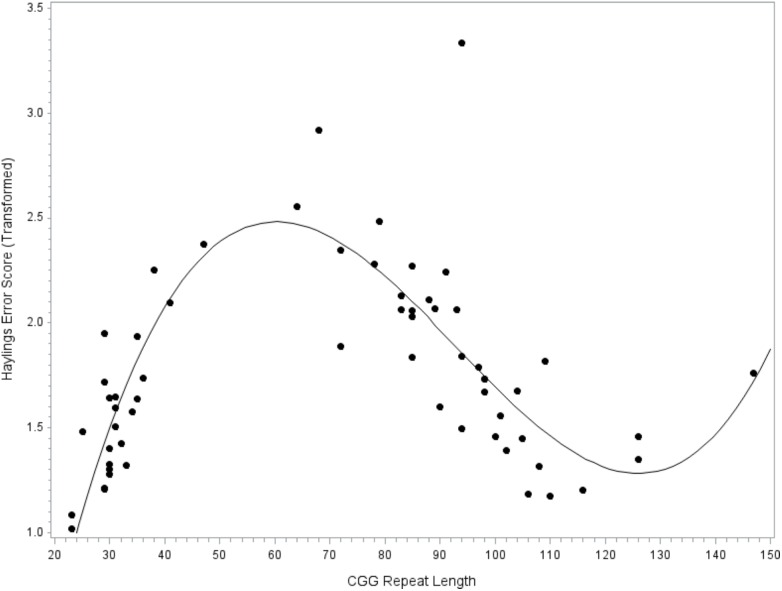
Relationship between verbal inhibition and CGG repeat length. Cubic association between CGG repeat length and inhibition skill. Model-predicted values are shown, controlling for age, education level, IQ, and allele-1 CGG size. A higher Hayling error score reflects poorer verbal inhibition skills.

The next model tested CGG repeat size as a predictor of verbal inhibition. Results indicated that the overall model did not account for significant variance in the Hayling error score, *F*(9,47) = 1.46, *p* = 0.191, *R*^2^= 0.22. However, the individual linear, quadratic, and cubic terms for CGG size did account for significant variance, with medium-to-large effect sizes (linear, *p* = 0.012, $\eta_{p}^{2}$ = 0.13; quadratic, *p* = 0.017, $\eta_{p}^{2}$ = 0.12; and cubic, *p* = 0.026, $\eta_{p}^{2}$ = 0.10); see **Figure 3**. The covariates did not contribute significantly to the model (all *p*’s > 0.232). To facilitate comparison with prior studies focused on the *FMR1* premutation, the model was repeated while including only individuals with premutation alleles. The overall models testing linear, quadratic, and cubic CGG predictors did not account for significant variance in verbal inhibition skills (all *p*’s > 0.419) and the individual terms for CGG repeat size were also not significant (all *p*’s > 0.122).

## Discussion

Building on emerging evidence that phenotypic variation may occur across the full range of CGG repeat size, this study adopted an individual-differences approach to investigate linguistic and cognitive phenotypes occurring across the continuous range of normal, intermediate, and premutation alleles. A robust curvilinear association between CGG length and language disfluency was detected, where low-normal alleles of about ≤25 repeats and mid-size premutation alleles of about 90–110 CGG repeats were associated with elevated risk. Disfluency was not associated with inhibition deficits, which challenges prior theoretical work and suggests that other factors, such as a primary language deficit, could account for elevated language disfluency in *FMR1-*associated conditions. The detected CGG-dependent patterns of language disfluency open a new line of inquiry into the role of *FMR1* gene function in language production processes, both in individuals with and without expanded CGG repeats on *FMR1.*

This study builds on the work of Sterling et al. (2013), who were the first to report increased disfluency in women with the *FMR1* premutation, but did not detect an association between disfluency and CGG repeat length within the premutation range. Our study incorporated a wider range of allele sizes and allowed for non-linear relationships, yielding new evidence of CGG-dependent changes in disfluency occurring across the full range of normal, intermediate, and premutation alleles. The curvilinear association between CGG repeat size and disfluency was robust and evident even after accounting for relevant confounds, including education level, age, and IQ. This finding suggests associations with CGG repeat size occurring across the continuum of normal, intermediate, and premutation alleles that has not been evident in prior studies using categorical classification of CGG size. However, appropriate circumspect is warranted as our sample was characterized by limited representation at low-normal and intermediate allele sizes and replication in a larger sample is needed to confirm the patterns observed here. The detected risk patterns for language disfluency were largely consistent with the existing literature. Within the range of premutation alleles, we found elevated language disfluency at mid-size repeats (∼90–110 CGG repeats) relative to the higher and lower ends of the premutation range. This finding adds language production difficulties to the growing list of clinical features that have increased severity within the mid-size premutation range, which also includes psychiatric and reproductive problems (Sullivan et al., 2005; Ennis et al., 2006; Allen et al., 2007; Roberts et al., 2009; Seltzer et al., 2012; Mailick et al., 2014a; Loesch et al., 2015).

A sharp increase in disfluency was also observed at the low end of the normal range, beginning at about 25 CGG repeats. Others have also reported increased risk for adverse phenotypes at low-normal CGG lengths (e.g., Weghofer et al., 2012; Mailick et al., 2014b), although there is little consensus as to where the boundary of low-normal occurs. Our data are consistent with Gleicher et al. (2010), who identified the low-normal range as occurring at ≤25 CGG repeats based on descriptive analyses of the allele sizes of 358 women. It is notable that the severity of language disfluency at low-normal CGG sizes was similar to that observed within the mid-size premutation range, suggesting the potential for shared phenotypes across low-normal and mid-premutation CGG lengths that may be further explored in future work. This pattern of phenotypic overlap was also reflected in our model testing inhibition skills. Historically, *FMR1* has been viewed as a gene with high importance for fragile X-associated conditions, but little attention has been paid to the relevance of *FMR1* for the general population. Our findings, consistent with other emerging reports (e.g., Mailick et al., 2014b, 2017; Adamsheck et al., 2017), underscore the relevance of *FMR1* at the population level. Findings suggest that normal variation in *FMR1* CGG repeat length relates to the language production skills of individuals within the general population. However, replication in a larger sample with better representation within the low-normal range is needed to confirm this intriguing finding.

It will also be critical, in future research, to conduct targeted methodological work aimed at determining the optimal analytic strategy to account for the presence of two alleles in females. We employed an analytic technique that focused on the larger allele as the primary predictor while covarying for the size of the smaller allele, which is the method that has been used most widely in the extant literature (Gleicher et al., 2009a,b; Lledo et al., 2012; Voorhuis et al., 2014; Schufreider et al., 2015). This strategy was successful in detecting robust CGG associations in the present study. However, there are other possible methods to account for the presence of two alleles, such as focusing on the allele that deviates further from 30 as the primary predictor, similar to the technique adopted by Mailick et al. (2017). There is no consensus on the field regarding the optimal analytic approach, and little to no methodological work has been conducted to delineate the strengths and weakness of these varied analytic strategies. It is notable that, by focusing on the larger allele, the participants who were characterized as having a low-normal allele in the present study were indeed homozygous for low-normal repeats on both alleles (i.e., by default, if the larger of the two alleles was in the low-normal range, then the smaller allele was of an equal or smaller size). This may have increased our ability to detect phenotypic variation associated with the low-normal range, and it is possible, and perhaps likely, that the detected patterns do not generalize to individuals who carry only one low-normal allele. Follow-up work contrasting phenotypes associated with homozygous vs. heterozygous low-normal alleles in females is needed to address this empirical question. The distinction between homozygous and heterozygous low-normal alleles is critical because, while only about 4% of females carry low-normal CGG repeats on both X chromosomes, heterozygous low-normal alleles are common, seen in about one-third of females (Kraan et al., 2018).

Although the curvilinear patterns suggested that disfluency was elevated at low-normal and mid-premutation CGG sizes, it is unlikely that the increased disfluency reached the level of clinical impairment. The average rate of disfluency at these alleles sizes was ∼9%, compared to a rate of ∼7% observed in the rest of the study cohort. In non-disordered adult speech, the average rate of disfluency has been reported at about 6% (Bortfeld et al., 2001), which is similar to the rate of 7% observed here. Normal speakers show substantial individual variation in the rate of disfluency, which is influenced both by context as well as speaker characteristics (e.g., gender, IQ), which were controlled in this study (Bortfeld et al., 2001; McDougall and Duckworth, 2017). Even when disfluencies do not reach clinical thresholds, the quantification of these features can lend insight into broader neurocognitive risk. For example, disfluencies have proven useful as a marker of early neurodegeneration and disease progression in clinical groups such as Alzheimer’s and Parkinson’s disease (McDowd et al., 2011; López-de-Ipiña et al., 2013; Tóth et al., 2015). As discussed by Sterling et al. (2013), it is possible that the presence of disfluencies within the context of the *FMR1* premutation could portend the later development of FXTAS; further research is needed to test this hypothesis.

Prior studies of the *FMR1* premutation have postulated that language disfluency marks executive dysfunction (Sterling et al., 2013; Movaghar et al., 2017). An aim of the present study was to test this assumption, which would inform the mechanisms underlying disfluency relevant to the *FMR1* premutation. We focused specifically on inhibitory aspects of executive function, given the theoretical ties between language production errors and poor inhibitory control (i.e., the Inhibition Deficit Hypothesis; Hasher and Zacks, 1988). Our data are at odds with the Inhibition Deficit Hypothesis, as the overall severity of language disfluencies was not associated with inhibition errors on the Hayling. We did observe a correlation between inhibitory control and a specific subtype of disfluencies: repairs. However, the direction of the association was contrary to the Inhibition Deficit Hypothesis, where individuals who exhibited *greater* difficulty on the verbal inhibition task were *less likely* to exhibit revisions or restarts in their language. Relatively few prior reports have directly tested associations between inhibition and disfluency, making it difficult to interpret this finding. The direction of effects detected here is opposite that reported by Engelhardt et al. (2013), who found that inhibition difficulties accounted for about a third of the variance in repair disfluencies in typically developing speakers. However, there are a number of methodological differences across studies that may account for the discrepant findings, such as the focus on a younger, mixed sex sample in the Engelhardt study, as well as the measurement of disfluency from a constrained sentence production task. Age, gender, and sampling context are all factors that have been shown to influence the rate of disfluency (Bortfeld et al., 2001; Shriberg, 2001). Additionally, Engelhardt and colleagues found that repair disfluencies were only associated with certain inhibition measures (the Stroop task but not the Stop task), suggesting that choice of inhibition measure may influence results; we used the Hayling Sentence Completion Task in the current study because prior research suggests that it is sensitive to premutation-associated inhibitory deficits (Kraan et al., 2014b). Overall, the role of individual differences in executive function have only begun to be explored with respect to language production, and research has yet to link the variance in executive skills with specific processes or sub-processes of the language production system. These findings highlight the need for more nuanced investigation into the mechanisms associated with language disfluency, both in the *FMR1* premutation as well as in the general population.

One possible interpretation of our findings is that language disfluencies do not arise from inhibitory deficits specifically, but nonetheless are influenced by other aspects of executive function. In general, a variety of executive control processes are necessary for fluent language production, such as planning, self-monitoring, and attention (Bosshardt, 2002; Turkstra et al., 2004; Schnadt and Corley, 2006; Felsenfeld et al., 2010). It may not be possible to trace disfluency back to a single executive domain. Additionally, the hypothesis that disfluency is a primary reflection of executive deficits may be misguided because it largely ignores the role of specific language processes in language production. For example, fluent language production relies heavily on language processes that support word-finding, such as phonological and lexical access (Levelt, 1992; Postma, 2000; Hartsuiker and Notebaert, 2010). Although cognitive-executive deficits have been researched more extensively in the *FMR1* premutation, the literature suggests that the specific language deficits may also occur in this group, such as abnormalities in semantic activation (Yang et al., 2014a,b), verbal recall (Shelton et al., 2017), verbal encoding (Hippolyte et al., 2014), and pragmatic language (Losh et al., 2012; Klusek et al., 2016). The present study raises the possibility that language-related deficits of the *FMR1* premutation may occur independent of an executive dysfunction phenotype.

The disfluency subtype analyses, which suggested that the observed *FMR1* CGG length associations were driven primarily by the occurrence of filled pause disfluencies, are also informative. Unlike repetition and repair disfluencies that are thought to arise as an unintentional by-product of difficulty within the language production system, filled pauses (e.g., “um,” “uh”) can be produced intentionally as *listener-oriented* signals that fulfill discourse-related pragmatic functions. For example, filled pauses can be used by the speaker to signal an upcoming delay, hold the conversational floor, and convey the speaker’s level of confidence (Clark, 1994; Brennan and Williams, 1995; Clark and Tree, 2002). Thus, filled pauses can serve as pragmatic markers, and some emerging evidence suggest that their occurrence is correlated with measures of autism symptom severity (Irvine et al., 2016). Pragmatic language difficulties and features of the broad autism phenotype have been documented in women with the *FMR1* premutation in a number of prior reports (Losh et al., 2012; Schneider et al., 2016; Klusek et al., 2017b,c, 2018). Follow-up studies are needed to explore the possibility that the filled pause disfluencies observed in the present study marked pragmatic, rather than executive, processes.

We are unable to draw strong conclusions regarding CGG-dependent changes in verbal inhibition. The individual predictors for linear, quadratic, and cubic CGG terms accounted for significant variance in verbal inhibition skills, with medium-to-large effect sizes, although this result should be interpreted with caution as the overall model was not significant. We may have been underpowered, perhaps due, in part, to the inclusion of several covariates that reduced degrees of freedom (inference was identical in a simplified model omitting covariates, although the model approached significance at *p* = 0.063; data not shown). Although the detected patterns are preliminary, they are consistent with emerging literature on CGG-dependent changes in behavioral inhibition. We detected poor inhibitory control at the low end of the premutation range, with inhibition skills improving with higher CGG repeat size until about 120 repeats (see **Figure 3**). This pattern mirrors the findings of Shelton et al. (2014), who found that ocular-motor inhibitory errors decreased linearly with increasing CGG size in women with the *FMR1* premutation. Notably, the Shelton et al. (2014) report was based on a truncated sample of women carrying premutation alleles of 61–102 CGG repeats, which may explain why a linear pattern fit the data. Our results were based on a wider range of premutation alleles (as high as 147 CGG repeats), which allowed us to detect a non-linear pattern where inhibitory errors began to increase at > 120 CGG repeats. Other reports have not detected associations between CGG size and inhibition, but have relied only on tests of linear association that may have obscured patterns (e.g., Kraan et al., 2014a,b). It will be important to test for non-linear associations in future research on *FMR1-*associated variation to clarify the inconsistencies in the literature. Another notable finding related to the inhibition data was that the patterns of CGG-associated risk differed across language disfluency and verbal inhibition. For instance, mid-size premutation alleles were associated with decreased risk for inhibition deficits but increased risk for disfluency. Additional research is needed to explore these preliminary findings, which may indicate mechanistic specificity or domain-specific effects.

Regarding intermediate or “gray zone” alleles, the results of the present study are tentative and replication is needed, as our sample was underrepresented within this range. Preliminary findings suggested that intermediate repeat sizes were associated with the lowest level of disfluency and the highest level of inhibition errors relative to CGG repeat sizes in the normal and premutation ranges. This study highlights intermediate alleles as an opportunity for future research. Prior investigations of intermediate alleles have focused primarily on movement disorders and reproductive function (e.g., Bodega et al., 2005; Debrey et al., 2016; Entezari et al., 2017; Wang et al., 2017) and findings are controversial, as not all reports indicate increased risk in these areas (Toft et al., 2005; Bennett et al., 2010; Kline et al., 2014). To our knowledge, no prior studies have investigated cognitive or linguistic phenotypes relative to intermediate alleles, which is a fruitful avenue of future investigation as intermediate alleles affect a significant proportion of the population (about 1:57 individuals; Cronister et al., 2008).

There are a number of future directions and considerations related to the findings of this study. First, replication in a larger sample with better representation across the full range of normal, intermediate, and premutation allele sizes will lend further credence to results. The representation of our study cohort was limited at some allele sizes, particularly within the low-normal, intermediate, and higher end of the premutation ranges. As previously discussed, this study underscores the need for methodological work to determine the most optimal analytic technique for accounting for the presence of two alleles in females, particularly with respect to the characterization of homozygous vs. heterozygous low-normal alleles. This work could also be extended through the incorporation of FMRP and *FMR1* mRNA indices in future studies, which may be more accurate predictors of *FMR1-*related phenotypes. It is also notable that our sample consisted of mothers who had children with developmental disorders (fragile X syndrome or ASD). The inclusion of mothers of children with ASD to represent normal/intermediate CGG repeat sizes was necessary to account for the potential impact of parenting stress on cognitive and linguistic performance across all participants. However, a limitation of this design is that it is unclear whether the cognitive and linguistic attributes of parents of disabled children are representative of the general population. For example, there is evidence that the chronic stress of parenting a child with a disability is linked with accelerated cognitive aging (Song et al., 2015) and therefore could theoretically also impact language production performance. Inclusion of an additional comparison group of mothers of typically developing children, or mothers of children with other intellectual or developmental disabilities, would have allowed us to better determine caregiver-related effects. Additionally, fragile X syndrome and ASD vary in their severity and presentation and it is possible that we were unable to completely account for differences in caregiver burden across the range of normal/intermediate and premutation alleles. Finally, our focus on inhibition as a correlate of disfluency was guided by theory, but inhibition represents just one of several cognitive-executive mechanisms likely involved in language production; incorporating a wider battery of executive tests, and well as other measures of language function, would have allowed us to better tease apart the contributions of specific executive and language skills on disfluency.

## Conclusion

This study adopted an individual differences approach to examine cognitive and linguistic correlates of *FMR1* CGG repeat size across the continuum of normal, intermediate, and premutation allele ranges. We detected CGG-dependent changes in language disfluency, with evidence of increased risk at mid-premutation and low-normal alleles. Disfluency was not associated with verbal inhibition deficits, highlighting the need to clarify the relative contributions of executive and linguistic processes in disfluency associated with the *FMR1* premutation. Overall, this study provides novel evidence suggesting a role of *FMR1* in language production skills that is observed across both individuals with and without CGG expansions on *FMR1.*

## Data Availability Statement

The data that support the findings are available from the corresponding author upon reasonable request.

## Author Contributions

JK conceived the study, led the data collection, analysis, and interpretation, and drafted the manuscript. AP led the disfluency coding. LA and JR provided guidance on study design, data collection, and data interpretation. MM and BT contributed to data analysis and interpretation. TA, FT, and AG contributed to the collection and interpretation of the genetic data. All the authors contributed to the interpretation of the results, critical revision of the manuscript, and read and approved the final manuscript.

## Conflict of Interest Statement

The authors declare that the research was conducted in the absence of any commercial or financial relationships that could be construed as a potential conflict of interest.
